# Diurnal Cortisol Slope Mediates the Association Between Affect and Memory Retrieval in Older Adults With Mild Cognitive Impairment: A Path-Analytical Study

**DOI:** 10.3389/fnagi.2020.00035

**Published:** 2020-02-21

**Authors:** Rainbow T. H. Ho, Ted C. T. Fong, Joshua C. Y. Yau, Wai Chi Chan, Joseph S. K. Kwan, Patrick K. C. Chiu, Linda C. W. Lam

**Affiliations:** ^1^Centre on Behavioral Health, The University of Hong Kong, Pok Fu Lam, Hong Kong; ^2^Department of Social Work and Social Administration, The University of Hong Kong, Pok Fu Lam, Hong Kong; ^3^Department of Psychiatry, The University of Hong Kong, Pok Fu Lam, Hong Kong; ^4^Department of Medicine, The University of Hong Kong, Pok Fu Lam, Hong Kong; ^5^Imperial College Healthcare NHS Trust, Charing Cross Hospital, London, United Kingdom; ^6^Department of Psychiatry, The Chinese University of Hong Kong, Shatin, Hong Kong

**Keywords:** diurnal cortisol rhythm, early dementia, episodic memory, mild cognitive impairment, subjective mood, structural equation model

## Abstract

**Background:**

Memory deficits are linked to dysfunctional HPA axis activity and negative affect in older adults. This study evaluated the mediating effect of the diurnal cortisol pattern on the relationship between affect and memory in older people with mild cognitive impairment (MCI).

**Methods:**

This longitudinal study recruited 189 Chinese older adults with MCI from elderly centers in Hong Kong. The participants completed assessments of affect, salivary cortisol, and digit spans at baseline; neurocognitive assessments on verbal fluency, memory retrieval, and digit spans at 6-month follow-up; and instrumental activities of daily living (IADL) at 1-year follow-up. Structural equation modeling examined the direct and indirect effects of negative affect on memory and IADL via diurnal cortisol pattern.

**Results:**

Controlling for covariates, negative affect significantly predicted flattened diurnal cortisol slopes (β = 0.17, *p* < 0.05) but not memory or IADL (*p* = 0.23 – 0.91) directly. Diurnal cortisol slopes negatively predicted memory retrieval (β = −0.20, *p* < 0.05), which in turn positively predicted IADL (β = 0.22, *p* < 0.01). The indirect effect from negative affect to IADL via cortisol slope and memory retrieval was significant and negative (αβγ = −0.05, 95% bootstrapped CI = −0.248 to −0.001).

**Discussion:**

The present study established certain temporal linkages among affect and cortisol slopes at baseline, memory retrieval at 6 months, and functional decline 1 year later in older adults with MCI. Flattened diurnal cortisol slopes might mediate the detrimental effects of negative affect on memory retrieval and functioning across 1 year.

## Introduction

Memory is a complex mental process involving the encoding, storage, and retrieval of information (Moscovitch, 1992). Mild cognitive impairment (MCI) is a transitional stage beyond the expected cognitive decline of normal aging to the initiation of pathological decline due to dementia (Petersen et al., 1999). The clinical hallmark of Alzheimer’s disease, the most common form of dementia, is a progressive decline in memory functioning in episodic, short-term, and working domains. The accelerated memory decline poses further risks for adverse health outcomes (Jahn, 2013).

The loss of cognitive function in MCI may cause psychological distress and negative affect together with possible mind-body interactions. Psychological distress has been associated with declines in episodic memory and executive functioning among older adults (Ferrari et al., 2004; Wilson et al., 2007). Studies (Kremen et al., 2012; Sindi et al., 2012) have found negative associations between negative affect and memory functioning in cognitively healthy older adults. A longitudinal study (Rapp et al., 2011) demonstrated that depressive symptoms were predictive of further cognitive decline in older adults with dementia. The etiological role of depression in poor cognitive outcomes has been advocated via multiple pathways (Butters et al., 2008).

The HPA axis is the major neuroendocrine system that stimulates cortisol production to regulate the physiological response to stress and other homeostatic systems (Magri et al., 2006). The diurnal cortisol pattern is typically represented by an early morning peak followed by a gradual decline throughout the day. Disruption of the diurnal cortisol rhythm has been linked with worse cognitive functioning (Beluche et al., 2010; Almela et al., 2014; Lau et al., 2017; Montoliu et al., 2018) and greater risk of cognitive decline (Ouanes and Popp, 2019) in older adults without cognitive impairment. The underlying pathological conditions that cause MCI could also affect the HPA axis activity.

Dysfunctions in the HPA-axis activities have been found to contribute to more rapid cognitive decline in older adults with MCI (Csernansky et al., 2006) and co-morbid depressive symptoms in patients with Alzheimer’s disease (Du and Pang, 2015).

Recent studies have evaluated the mediating role of cortisol between personality traits and cognitive impairment (Ouanes et al., 2017) and between acute stressors and cognitive flexibility (Gabrys et al., 2019). Measurable features of the HPA axis activities include mean diurnal cortisol level in biofluids such as saliva, and the diurnal cortisol slope (Adam and Kumari, 2009). Dysregulation of HPA-axis activity may manifest in the form of flattened cortisol slopes, which may be caused by lower morning cortisol and/or higher evening cortisol levels. As the cortisol slope is regarded as reflecting resilience to stressful situations in daily life (Miller et al., 2007), it was of interest to investigate this specific parameter in conjunction with affect as a latent variable in the context of MCI.

Flattened diurnal cortisol slopes are associated with, or even caused by, emotion and stress in MCI subjects. A flattened diurnal cortisol slope has been associated with subjective memory complaints in older people without cognitive impairment (Peavy et al., 2013) and with cognitive impairment (Fiocco et al., 2006). Adam et al. (2017) have advocated a potential mediating role for flattened diurnal cortisol slopes in between psychosocial stress and a wide range of mental and physical health outcomes. Nevertheless, existing studies have not concurrently evaluated the inter-relationships among salivary cortisol, affect, and memory functioning in older adults with MCI. The potential etiological role of diurnal cortisol slope in between affect and memory functioning remains to be determined.

The present study aimed to examine the relationships among affect, salivary cortisol, memory, and daily functioning in older adults with MCI using structural equation modeling. Structural equation modeling is a comprehensive data analytic approach to evaluate *a priori* hypotheses about the relationships among observed and latent variables (Mueller and Hancock, 2018). Its use can separate the true common variance among the indicators from the error variance and provide more precise estimates (Bollen and Pearl, 2013). Given the role of emotion in establishing episodic memories (Mather, 2007; Dere et al., 2010) and the pivotal role of the HPA axis in the stress response, this study explored the mediating role of diurnal cortisol pattern by evaluating the indirect effects of affect as latent variable on memory and functioning via cortisol.

## Materials and Methods

### Study Design and Participants

The study sample included 189 Chinese older adults with MCI who were recruited from elderly daycare centers in Hong Kong from October 2015 to December 2016. This longitudinal study conducted various assessments at three waves: affect, salivary cortisol, and digit spans at baseline (Time 1); neurocognitive assessments on verbal fluency, memory retrieval, and digit spans at 6-month follow-up (Time 2); and IADL at 1-year follow-up (Time 3). This timeframe made it possible to evaluate the lagged effects from the predictors to memory and daily functioning at 6-month intervals. The timeframe was chosen with reference to the regular 6-month reevaluation period for individuals with MCI to monitor their disease’s progression.

The criteria for inclusion were being aged 65 or above, a diagnosis of cognitive impairment with a Clinical Dementia Rating of 0.5 or 1 (Hughes et al., 1982), and the ability to understand Cantonese. The exclusion criteria were concurrent major psychiatric disorder requiring hospitalization, the use of steroid medication, and severe illness that could cause substantial health deterioration. The study was carried out in accordance with the recommendations of the Declaration of Helsinki. The participants and their guardians were informed of the study objectives and procedures before providing written informed consent. The protocol was approved by the Human Research Ethics Committee of the University of Hong Kong.

The mean age of the study sample was 78.9 years (SD = 8.1) and the majority of the participants were female (82%), community-dwelling (78.8%), and had received at most 10 years of education (66.5%). Fourteen participants dropped out of the study at Time 2 and 18 at Time 3. Reasons for dropout included loss of contact or refusal to join the follow-up assessments. These dropouts (*N* = 32) did not differ significantly (*p* = 0.08 – 0.80) from the completers (*N* = 157) in terms of the baseline variables.

### Measurements

#### Negative Affect

Depressive symptoms were evaluated at Time 1 as the sum of four dichotomous items on the GDS (Cheng and Chan, 2005). Sample items included “Do you feel happy most of the time?” and “Do you feel that your life is empty?” The Visual Analog Mood Scale (Folstein and Luria, 1973) was used at Time 1 to measure participants’ positive mood (three items: relaxed, energetic, and delighted) and negative mood (five items: worried, nervous, angry, tired, and anxious) over the past week. The scale measures these feelings on an 11-point scale from 0 to 10. This scale has been used as a reliable assessment of mood among older people with Alzheimer’s disease (Scherder and Bouma, 2000). In the present study, acceptable reliability was found for the GDS (α = 0.64), positive mood (α = 0.77), and negative mood (α = 0.81).

#### Cortisol

At Time 1, the participants collected saliva samples using cotton salivette tubes at home five times on 1 weekday: wake-up (Sample 1), 1 h after wake-up (Sample 2), noon (Sample 3), late afternoon at 5 pm (Sample 4), and evening at 9 pm (Sample 5), i.e., corresponding to wake-to-bed slope (Adam et al., 2017). Previous studies (Lai et al., 2010; Ho et al., 2013) have demonstrated acceptable intra-individual stability and no significant difference between cortisol measures collected over two consecutive days. Written instructions and reminder notes were provided to assist the collection of the saliva samples. The participants were instructed to collect the first sample immediately after wake-up, record the sample collection times on a daily log, and avoid food consumption and exercise 30 min prior to sample collection. Instruction sheets and briefing sessions were provided to their caregivers to improve adherence to the sampling times.

The collected salivette tubes were kept frozen in the laboratory and cortisol levels were determined after thawing and centrifugation using an ELISA kit (Salimetrics, State College, PA, United States). The intra-assay and inter-assay variation was less than 10%. Of the 945 collected saliva samples, 855 (90.5%) provided valid cortisol values. The mean collection times of the saliva samples were 06:05 h (SD = 62 min), 07:09 h (SD = 75 min), 11:52 h (SD = 37 min), 16:55 (SD = 44 min) and 20:58 h (SD = 35 min), respectively. The current protocol of collecting five saliva samples per day has been shown to be more reliable than collecting plasma samples, with the added advantages of being non-invasive and stress-free (Adam et al., 2017). The raw cortisol values were used to derive the mean cortisol and the diurnal cortisol slope.

#### Memory

Four aspects of memory functioning were evaluated: episodic, verbal, short-term, and working memory. The Fuld Object Memory Evaluation (Ho et al., 2018b) was used to assess the participants’ memory and verbal fluency at Time 2. Detailed procedures for administering this neurocognitive test are available in Fuld et al. (1990). In brief, participants were instructed to identify and memorize 10 unrelated everyday items before completing five rapid verbal fluency tasks. They were asked to recall the 10 items within 60 s in between the five verbal fluency tasks (i.e., 50 trials in total). The memory retrieval score is the total number of items correctly recalled over the five recall trials, with a possible range from 0 to 50. Verbal fluency refers to the composite score of the number of objects named in the five verbal fluency tasks on categories such as animals, fruits, and vegetables. Memory retrieval and verbal fluency represent long-term episodic memory and verbal memory, respectively.

The Digit Span Test (Leung et al., 2011) was used to measure forward and backward digit span at Time 1 and Time 2. In this test, the participants listened to a sequence of numbers that increased in length from two to nine digits, with two trials for each sequence length. They were instructed to memorize the sequence and repeat the digits in the same (forward) or reverse (backward) order. The test ended when the respondent failed both trials of the same length. Forward and backward digit span scores denote the effect of short-term memory and working memory, respectively, on the maintenance and manipulation of information. Both cognitive tests were administered by four trained raters with acceptable inter-rater reliability (intraclass correlation coefficients >0.70).

#### Functional Outcome

At Time 1 and Time 3, the IADL scale (Tong and Man, 2002) was used to assess daily functional skills such as shopping, use of the telephone, doing laundry, taking medications, taking public transport, and handling finances. Participants’ ability to perform these tasks was evaluated via self-report and information from their caregivers. A previous study (Trigg et al., 2007) demonstrated the validity of using self-reported quality of life assessments with people with MCI. The total IADL score ranges from 0 to 18 with higher scores indicating greater independence in daily functioning. This scale showed excellent reliability (α = 0.90) at Time 1.

### Conceptual Model

**FIGURE 1:**
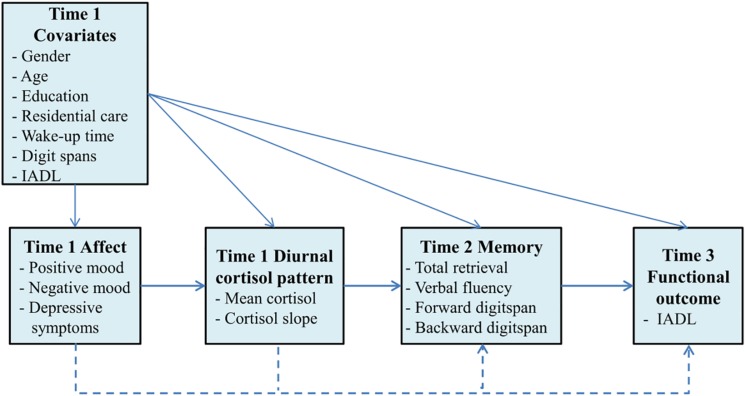
Conceptual model of affect, diurnal cortisol pattern, memory, and functional outcome across the 1-year period. The pathways of the cascading path model are denoted by solid lines and the combined path model contains additional direct effects (dashed lines) from affect to memory and functioning, and from diurnal cortisol pattern to functional outcome.

Figure 1 depicts the conceptual model of the present study. Negative affect was modeled as a latent predictor variable based on the observed indicators of depression, positive mood, and negative mood at Time 1. Indicators of diurnal cortisol pattern (mean cortisol and diurnal cortisol slope) at Time 1 were proposed as potential mediator variables. Memory functioning at Time 2 was hypothesized as the outcome variable, and functional outcome at Time 3 was considered the distal outcome. The cascading path model (solid lines) examined the indirect effects of negative affect on memory and IADL via diurnal cortisol patterns, and the combined path model included direct effects (dashed lines) from negative affect to memory and IADL. Demographic characteristics (age, gender, education, care setting, and wake-up time) and baseline digit span and daily functioning measures were included as control variables in the model.

### Data Analysis

Path analysis was conducted using structural equation modeling in Mplus 8.2 (Muthén and Muthén, 2017) to examine the direct and indirect effects from negative affect as latent variable to memory functioning and functional outcome via diurnal cortisol pattern. Structural equation modeling involves the steps of model formulation, parameter identification and estimation, data-model fit evaluation, and potential model modification (Wang and Wang, 2019). Robust maximum likelihood estimation was used to account for non-normality.

Cortisol analysis was based on 838 valid samples (98.0%) after removing 17 outliers that deviated substantially (>3 standard deviations) from the mean. The mean cortisol level was computed by dividing the cortisol area-under-the-curve by the time elapsed from Sample 1 to Sample 5. The diurnal cortisol slope was computed via latent growth modeling on a latent time basis. Sensitivity analysis was performed by repeating the calculation of the mean cortisol and diurnal cortisol slope with the inclusion of the 17 outliers.

The indirect effects of the Time 1 predictors on Time 3 functioning via Time 2 memory were evaluated using 10,000 bootstrapping samples to account for the non-normal distribution. The indirect effects were statistically significant if the 95% bootstrapped confidence intervals did not include zero. Given the potential reciprocal effects from diurnal cortisol pattern to affect, we specified an alternative structural equation model with a reverse directional path from Time 1 diurnal cortisol pattern to Time 1 negative affect for comparison. In this alternative model, we tested the indirect effect from Time 1 diurnal cortisol pattern to memory functioning and functional outcome via Time 1 negative affect.

Model fit was evaluated using the following criteria: non-significant χ^2^ (*p* > 0.05), CFI ≥ 0.95, and root mean square error of approximation (RMSEA) and SRMR ≤ 0.06. Model comparison was based on the chi-square difference test. The standardized path coefficients indicated the effect size with values of 0.1, 0.3, and 0.5 denoting small, moderate, and large magnitudes, respectively (Cohen, 1988). Missing data were handled via full information maximum likelihood under the missing-at-random assumption (Schafer and Graham, 2002).

## Results

### Sample Characteristics

Table 1 presents the descriptive statistics of the main study variables. At Time 1, the participants reported moderate levels of positive mood and daily functional skills and low levels of depressive symptoms and negative mood. At Time 2, the participants successfully retrieved around 7.4 out of the 10 items (SD = 2.5) over the five recall trials in the Fuld Object Memory Evaluation. Forward and backward digit spans remained stable (*r* = 0.58 – 0.63) across Time 1 and Time 2 with means of around 6.6 (SD = 1.5) and 3.3 (SD = 1.6), respectively. The sample showed a moderate decline (Cohen *d* = 0.46, *p* < 0.01) in IADL over 1 year. Positive correlations (*r* = 0.17 – 0.62, *p* < 0.05) were found among the memory indicators on verbal fluency, retrieval, and digit spans at Time 2. Time 3 IADL was negatively associated with diurnal cortisol slope (*r* = −0.19, *p* < 0.01).

**TABLE 1:** Descriptive statistics and bivariate correlations among the main study variables.

		Mean	SD	1	2	3	4	5	6	7	8	9	10	11	12
1	Time 1 Depressed symptoms	0.94	1.15												
2	Time 1 Negative mood	3.45	2.06	0.51**											
3	Time 1 Positive mood	5.56	2.01	−0.50*	−0.37**										
4	Time 1 Mean cortisol	4.64	2.55	–0.05	–0.12	0.00									
5	Time 1 Diurnal cortisol slope	–4.35	2.09	0.11	0.13*	–0.08	−0.62**								
6	Time 1 Forward digit span	6.56	1.45	0.03	−0.17*	–0.06	0.02	–0.10							
7	Time 1 Backward digit span	3.27	1.58	–0.05	0.09	–0.01	0.05	–0.13	0.36**						
8	Time 1 IADL	13.3	4.91	–0.07	0.13	0.16*	0.01	–0.11	0.33**	0.41**					
9	Time 2 Total retrieval	37.1	12.4	0.02	0.11	0.08	–0.10	–0.10	0.17*	0.32**	0.51**				
10	Time 2 Verbal fluency	28.0	8.41	0.03	−0.16*	0.06	0.00	–0.07	0.28**	0.43**	0.59**	0.62**			
11	Time 2 Forward digit span	6.74	1.48	–0.01	0.10	–0.04	–0.04	0.05	0.58**	0.29**	0.29**	0.17*	0.34**		
12	Time 2 Backward digit span	3.29	1.59	–0.05	0.03	0.00	0.06	–0.10	0.42**	0.63**	0.34**	0.26**	0.40**	0.50**	
13	Time 3 IADL	11.2	5.90	–0.03	−0.18*	0.07	0.08	−0.19**	0.28**	0.37**	0.70**	0.57**	0.56**	0.30**	0.40**

**p < 0.01; *p < 0.05.

The mean cortisol levels of the five saliva samples were 6.92 nmol/L (SD = 5.63), 8.72 (SD = 7.15), 4.55 (SD = 4.45), 3.57 (SD = 3.73), and 2.81 (SD = 2.99), respectively. The five cortisol samples showed significant positive correlations (*r* = 0.24 – 0.48, *p* < 0.01) between adjacent samples but not between samples with two or more lags (*r* = −0.15 to 0.17, *p* > 0.05). The latent basis growth model provided an adequate fit to the cortisol measures with χ^2^(5) = 8.11, *p* = 0.15, CFI = 0.94, and RMSEA = SRMR = 0.06. The mean cortisol level throughout the day was 4.64 nmol/L (SD = 2.55) and the average diurnal cortisol slope was −4.35 nmol/L/day (SD = 2.09). The mean cortisol and diurnal cortisol slope were negatively correlated (*r* = −0.62, *p* < 0.01). The sensitivity analysis with the 17 outliers included showed similar results for the diurnal cortisol pattern.

### Structural Equation Model Estimates From Affect to Cortisol

Table 2 displays the fit indices for the structural equation models. Both the combined and cascading models of the paths from affect to diurnal cortisol pattern showed acceptable fits to the data with CFI > 0.95, and RMSEA and SRMR < 0.05. The non-significant result (*p* = 0.41 > 0.05) in the chi-square difference test favored the cascading path model as it was more parsimonious with no reduction in the model fit. In the cascading path model, the latent factor of negative affect showed substantial loadings on the three indicators (λ = 0.64 – 0.78, *p* < 0.01) and acceptable composite reliability (Ω = 0.68). Table 3 lists the significant paths from the baseline control variables to the main study variables in the cascading path model.

**TABLE 2:** Model fit indices of the structural equation models on affect and cortisol.

		Chi-square test			Goodness-of-fit indices			Chi-square difference test		
Direction (affect-cortisol)	Type	χ^2^	*df*	*p*	CFI	RMSEA	SRMR	△χ^2^	△*df*	△*p*
Affect → Cortisol	Combined	55.4	38	0.034	0.98	0.049	0.039			
	Cascading	62.4	45	0.044	0.98	0.045	0.041	7.22	7	0.41
Cortisol → Affect	Combined	55.4	38	0.034	0.98	0.049	0.039			
	Cascading	70.4	49	0.024	0.97	0.048	0.042	15.1	11	0.18

*χ^2^, chi-square; df, degree of freedom; CFI, comparative fit index; RMSEA, root-mean-square error of approximation; SRMR, standardized root mean square residual; △, change.*

**TABLE 3:** Effects of baseline control variables on main study variables in the cascading path model.

Control variable	Dependent variable	B (SE)	β	Control variable	Dependent variable	B (SE)	β
Age	Affect	−0.04 (0.01)**	−0.34	Wake-up time	Mean cortisol	−0.52 (0.22)*	−0.25
	T3 IADL	−0.15 (0.05)**	−0.21		T2 Total retrieval	−0.25 (0.07)**	−0.25
Female	T2 Verbal fluency	0.37 (0.13)**	0.17	T1 Forward digit span	T2 Forward digit span	0.55 (0.08)**	0.53
	T3 IADL	−1.82 (0.74)*	−0.12		T2 Backward digit span	0.20 (0.06)**	0.18
Residential care	T3 IADL	−3.54 (0.74)**	−0.24	T1 Backward digit span	T2 Backward digit span	0.48 (0.08)**	0.47
Education	Mean cortisol	0.34 (0.16)*	0.19	T1 ADL	T2 Total retrieval	0.09 (0.02)**	0.35
	T2 Verbal fluency	0.14 (0.04)**	0.24		T2 Verbal fluency	0.07 (0.01)**	0.43
	T2 Backward digit span	0.16 (0.07)*	0.15		T3 IADL	0.44 (0.08)**	0.37

B, unstandardized estimate; β, standardized estimate; T1/T2/T3, Time 1/2/3; only statistically significant effects are shown. **p < 0.01; *p < 0.05.

**FIGURE 2:**
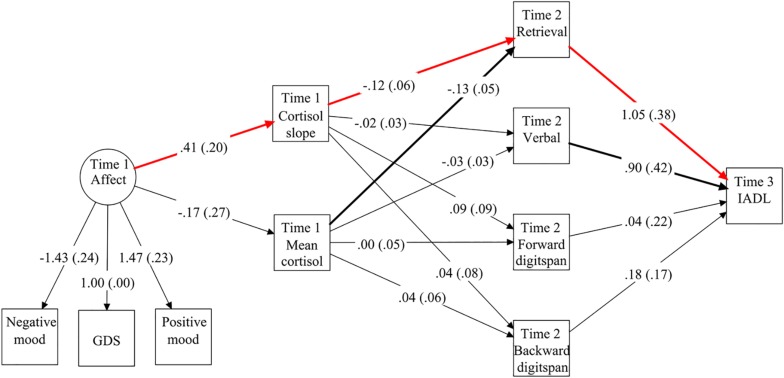
Unstandardized path coefficients in the structural equation model, with significant paths among the main study variables highlighted in bold. The red paths denote the indirect effect from negative affect to IADL via diurnal cortisol slope and memory retrieval. Standard errors are shown in parentheses. To simplify the presentation, the figure does not include the demographic and baseline control variables or the residual covariances among the dependent variables.

Figure 2 displays the unstandardized regression estimates for the main study variables in the cascading path model. Controlling for the covariates, negative affect significantly and positively predicted diurnal cortisol slope (*B* = 0.41, SE = 0.20, *p* < 0.05, β = 0.17) but not mean cortisol level (*B* = −0.17, SE = 0.27, *p* = 0.53, β = −0.06). Both mean cortisol level (*B* = −0.13, SE = 0.05, *p* < 0.05, β = −0.27) and diurnal cortisol slope (*B* = −0.12, SE = 0.06, *p* < 0.05, β = −0.20) negatively predicted Time 2 memory retrieval. However, their effects on verbal fluency and digit spans were not statistically significant (*p* = 0.28 – 0.95). Time 3 IADL was positively predicted by memory retrieval (*B* = 1.05, SE = 0.38, *p* < 0.01, β = 0.22) and verbal fluency (*B* = 0.90, SE = 0.42, *p* < 0.05, β = 0.13) but not digit span (*p* = 0.31 – 0.87). The proportion of explained variance ranged from 40.4% to 60.8% for depression, negative mood, and positive mood, 5.9% to 10.2% for diurnal cortisol pattern, 37.7 to 48.9% for Time 2 memory indicators, and 71.9% for Time 3 IADL.

### Indirect Effects From Affect to Memory Function and IADL via Cortisol

In the combined path model, the direct effects of negative affect on memory function (*p* = 0.23 – 0.91) and IADL (*B* = −0.06, SE = 0.46, *p* = 0.89, β = −0.01) were not statistically significant. Neither mean cortisol (*B* = 0.20, SE = 0.20, *p* = 0.32, β = 0.09) nor cortisol slope (*B* = −0.11, SE = 0.18, *p* = 0.54, β = −0.04) showed a significant direct effect on IADL. There was a significant and negative indirect effect (αβγ = −0.050, 95% bootstrapped CI = −0.248 to −0.001) from Time 1 negative affect to Time 3 IADL via Time 1 diurnal cortisol slope and Time 2 memory retrieval. The remaining indirect effects via mean cortisol or other memory functions were not statistically significant, with the 95% bootstrapped CIs including zero.

### Alternative Structural Equation Models From Cortisol to Affect

Regarding the alternative structural equation models from diurnal cortisol pattern to affect, the cascading path model was more parsimonious and the model fit was no worse than for the combined path model (*p* = 0.18 > 0.05) (Table 2). In this cascading path model, neither mean cortisol nor diurnal cortisol slope significantly (*p* > 0.05) predicted the latent factor of negative affect at Time 1. Similarly, Time 1 negative affect did not significantly predict any of the memory functioning measures (*p* = 0.26 – 0.89) at Time 2. None of the indirect effects from diurnal cortisol pattern to Time 3 IADL via Time 1 negative affect and Time 2 memory functioning were statistically significant, with all 95% bootstrapped CIs including zero.

## Discussion

This longitudinal study is the first to evaluate the inter-relationships among affect, the HPA axis, memory functioning, and IADL in Chinese older adults with MCI. Greater negative affect was found to predict flattened diurnal cortisol slopes, but the reverse effect was not found in the alternative structural equation model. Despite the use of concurrent measures at baseline, these findings appear to support a causal direction from affect to neuroendocrine functioning. In line with previous findings among cognitively healthy older adults (Gerritsen et al., 2011; Stawski et al., 2011; Johar et al., 2015), flattened diurnal cortisol slopes were linked with diminished memory retrieval and worsening daily functioning. In this study, better emotional well-being in the older adults with MCI appears to predict better functioning 12 months later by maintaining the diurnal cortisol slope and memory retrieval.

The current study revealed a significant and negative indirect effect of negative affect on memory retrieval and functional skills via diurnal cortisol slopes among the sample of older adults with MCI. This finding suggests that cortisol has a bridging role between functioning in the emotional and cognitive domains. HPA axis activity could be an underlying mechanism by which prolonged emotional distress leads to memory retrieval deficits (Wolf, 2017) and subsequent functional decline in daily living. Our findings correspond to the established linkage between flattened diurnal cortisol slopes and physical and mental health outcomes in general (Adam et al., 2017).

The non-significant indirect effects from affect to memory and IADL via mean cortisol could partly be due to the high variance in the five salivary cortisol samples. Salivary cortisol has been shown to be susceptible to multiple sources of variance from numerous confounders and medical conditions (Hellhammer et al., 2009). Therefore, the diurnal cortisol slope might be a more sensitive indicator of HPA axis activity than the mean cortisol level in stress modulation. Despite the significant indirect effects for memory retrieval, no such indirect effects were found for functioning in other (verbal, short-term, and working) domains. This discrepancy could be explained by the potential role of emotion in establishing episodic memories (Mather, 2007; Dere et al., 2010; Rimmele et al., 2015) and suggests greater clinical relevance between episodic memory retrieval and psychoendocrinological factors.

### Study Strengths

There were several strengths in the present study. The first was the longitudinal design with an adequate sample size over the 1-year period. Second, the structural equation model included affect as a latent predictor measured by depression, positive mood, and negative mood. Structural equation modeling is an innovative and powerful analytic approach because it helps to account for measurement errors in the mood indicators and enables the combined effects of these observed indicators to be assessed (Kremen et al., 2012). Although the present longitudinal design was of necessity preliminary and limited with repeated measurements conducted only for digit spans and IADL, this analytic approach was validated in demonstrating significant indirect associations among the latent affect, cortisol measures, memory retrival and IADL. Third, the study variables were measured from multiple sources (the participants, their caregivers, and professional raters) through various means (objective cortisol biomarkers, self-report measures of affect and IADL, and cognitive assessments). The inclusion of both subjective and objective data mitigates common method bias and improves the credibility of the results.

### Study Limitations

The study also had several limitations. First, it did not explicitly distinguish between different types of dementia (Alzheimer’s disease, vascular dementia, frontotemporal dementia, and dementia with Lewy bodies). Given the potential etiological role of cortisol in Alzheimer’s disease (Du and Pang, 2015), the dementia subtype might modulate the interpretation of the results. Future larger-scale studies should conduct multi-group structural equation modeling to explore the inter-relationships among the study variables across dementia subtypes. Second, although the present study primarily hypothesized a causal effect from affect to memory functioning, it is plausible that memory decline could worsen the emotional state of older people. The reciprocal effect from memory decline to affect could thus not be ruled out and should be elucidated in further studies.

Third, the self-reported time of awakening and saliva collection was subject to recall bias given the participants’ cognitive impairment. Assessment of the cortisol awakening response requires saliva sampling accuracy (Stalder et al., 2016) that could not be precisely captured under the current sampling scheme. Additional saliva samples should be collected at 15 and 30 min after wake-up to elucidate the role of the awakening response in the structural equation model. Instead of focusing on the diurnal cortisol slope, future research could alternatively examine the mediating role of morning and evening cortisol levels. Fourth, the study did not control for other potential confounders such as sleep disturbance, early life adversity, recent stress events, and social interactions (Kremen et al., 2012). These unmeasured confounders may have biased the direct and indirect effects from affect to the outcomes. Fifth, the diurnal cortisol pattern was assessed via five salivary cortisol samples on a single day only. The failure to take into account day-to-day fluctuations may have reduced the effect sizes and significance of associations.

### Clinical Implications

The current study revealed an indirect but clinically salient link between negative affect and memory retrieval via diurnal cortisol slopes in older adults with MCI. Most if not all older adults with MCI are at risk of developing Alzheimer’s disease, the most common type of dementia. Given the established role of apolipoprotein E as a risk factor for Alzheimer’s disease (Gerritsen et al., 2011), future studies could investigate the links between this genetic marker and well-being in the emotional and cognitive domains. The present findings contribute to the literature and highlight the importance of addressing the biopsychosocial needs of these individuals in various domains; and early intervention on prevention of negative affect might be helpful to delay or alleviate further deterioration in their cognitive abilities and functional decline in the pre-dementia stages (Robinson et al., 2015).

Therapies and complementary treatments should be formulated to enhance the multidimensional functioning of older adults and delay the progression from MCI to dementia. Holistic interventions should be developed based on the biopsychosocial model and the person-centered approach (Olanrewaju et al., 2015). These interventions could integrate useful elements from the physical, cognitive, and psycho-behavioral domains. For instance, physical exercise could improve muscle health and cognitive stimulation therapy could help preserve memory functioning or slow down the rate of deterioration. Reminiscence therapy, music therapy (Ho et al., 2018c), and dance–movement therapy (Ho et al., 2018a) have shown beneficial effects such as ameliorated behavioral and psychological symptoms and improved mood and functioning.

## Conclusion

For older adults with cognitive impairments, the inter-play among emotional, neuroendocrine, and cognitive domains has proven to be a complex and problematic issue to date. Utilizing the conceptual framework of the structural equation modeling approach, the current study revealed for the first time a pathway from affect to retrieval and daily functioning, via diurnal cortisol pattern, in older adults with MCI. The present findings may help elucidate the psychoendocrinological determinants of memory functioning and provide potentially useful insights into the mediating role of cortisol on the longitudinal effect of emotional well-being on memory functioning.

## Data Availability Statement

The datasets generated for this study are available on request to the corresponding author.

## Ethics Statement

The study was carried out in accordance with the recommendations of the Declaration of Helsinki. The participants and their guardians were informed of the study objectives and procedures before providing written informed consents. The protocol was approved by the Human Research Ethics Committee of the University of Hong Kong.

## Author Contributions

RH designed the study, supervised the study implementation, and formulated the research question. TF performed the literature review, conducted the data analysis, and interpreted the results. JY collected the data and performed assays of the salivary cortisol samples. WC and LL contributed to the conception and design of the study. JK and PC facilitated the acquisition of data. All co-authors participated in the writing of the manuscript.

## Conflict of Interest

The authors declare that the research was conducted in the absence of any commercial or financial relationships that could be construed as a potential conflict of interest.

## Abbreviations

CFI: comparative fit index
ELISA: enzyme-linked immunosorbent assay
GDS: geriatric depression scale
HPA: hypothalamic-pituitary-adrenal
IADL: instrumental activities of daily living
RMSEA: root-mean-square error of approximation
SRMR: standardized root mean square residual.
